# 

**Published:** 1887-10-08

**Authors:** 


### NOTICE TO ADVERTISERS.

THE scale of charges for small prepaid Advertisements—Situations Wanted, Situations Vacant, etc., etc., is as follows :—

| | s. | d. |
|---|---|---|
| One insertion of three lines | 1 | 0 |
| Three „ „ „ | 2 | 6 |
| Per line afterwards | 0 | 6 |

*A line averages ten words.*

All copy for Advertisements must be sent to A. P. WATT, 2, PATERNOSTER SQUARE, E.C., by first post on the Tuesday *preceding* publication.

### NOTICE TO CORRESPONDENTS.

All correspondence must be written on one side of the paper only.
We cannot undertake to return MSS. not used.
**Communications**, whether intended for insertion or for private information, must be authenticated by the names and addresses of the writers, not necessarily for publication.
**Local papers** containing reports or news paragraphs **should be marked.**
It is especially requested that early intelligence of **local events of interest**, or which it may be thought desirable to bring under notice, may be sent direct to the Editor.
Communications respecting editorial matters should be addressed to the Editor, Norfolk House, Norfolk Street, Strand, London, W.C.

⁂ AMUSEMENTS WITH PRIZES FOR ALL AGES, will be found on page iv of this week's issue.

### RESULTS OF FOURTH QUARTERLY PRIZE COMPETITIONS.

ANOTHER quarter has come to an end, and the results of the *Puzzle* and *Letter* Competitions are published to-day. The contest has again been a very close one, and I am glad that *new* as well as *old* friends have gained prizes. It is hoped that each competitor will persuade one or more friends to enter the lists for the ensuing Competitions, which from the heading will be seen, are varied this Quarter.

PUZZLES.

*First Prize*, 30*s*., won by UMSLOPOGAAS, score 48. (Master S. H. Anstey, 25, Dornton Road, Balham, S.W., aged 12.)

*Second Prize*, 20*s*., won by SCROOGE, score 47. (Miss Constance L. Mills, 7, Charles Street, Pendleton, Manchester, aged 13.)

*Third Prize*, 10*s*., won by A. C. K., score 46. (Master Arthur Clifton Kelly, Ellesmere, Gratwicke Road, Worthing, aged 10.)

*Fourth Prize*, value 5*s*., is a tie between MOUNT EDGCUMBE (Master Charles Maddock, Luccombe House, Redland Green, Redland, Bristol, aged 14½), and TOBY (Master Stephen Reginald Bradley, 47, Mimosa Street, Fulham, S.W.).

LETTERS.

Here we have another tie between SOCRATES (Master Frank Craig, 13, Burma Road, Green Lanes, Stoke Newington, aged 13), and TOBY (Master Stephen R. Bradley, 47, Mimosa Street, Fulham, S.W.). I therefore divide the prize, 10*s*., between them.

NEATNESS.

The prize of 5*s*. for neatest answers to puzzles has been gained by Master A. G. S. McCulloch, 11, Highbury Quadrant, London, N., aged 11.

### QUESTIONS AND ANSWERS.

All questions should be addressed to the Editor, Norfolk House, Norfolk Street, Strand, W.C.

C. D. "CONSTANT READER."—We sympathise very much with your dyspeptic troubles, but on no account can we recommend any particular physician.

### The In- and Out-Patients' Hospital Diary.

This Diary is so arranged as to make some portion of it appear every week, and the whole in each monthly part. It has been very difficult to compile, and corrections will at all times be gratefully received. It has been suggested that similar information about the larger Provincial and Scotch Hospitals, would be useful to many readers. We should be glad to hear if this is felt to be the case.

### I.—GENERAL HOSPITALS.

| Hospitals and Terms of Admission. | Physicians, Surgeons, and Medical Officers, with Days and Hours of Attendance. |
|---|---|
| **Charing Cross Hospital**, West Strand, W.C. Sec., Mr. A. E. Reade<br>Urgent cases are immediately admitted as in-patients. Subscriber's letter expected in other cases<br>Out-patients admitted without subscriber's letter first time; for continued attendance they must be obtained from the Secretary | *In-Physicians*, Drs. Pollock, M. Th.; Green, W. S.; Bruce, Tu. F. Hour, 1.30<br>*In-Surgeons*, Messrs. Barwell, M. Th.; Bellamy, Tu. F.; Bloxam, W. S. Hour, 2<br>*Out-Physicians*, Drs. Wilcocks, M.Th.; Abercrombie, Tu. F.; Lubbock, W. S.; Murray, W. S. Hour, 1.30<br>*Out-Surgeons*, Messrs. Cantlie, M. Th.; J. H. Morgan, Tu. F.; Stanley Boyd, W. S. Hour, 1.30 |
| **"Dreadnought" Hospital** (Seamen's Hospital Society), Greenwich, S.E. Sec., Mr. P. J. Michelli<br>Entirely free to seamen and to all accident cases | *In- and Out-Physicians*, Drs. Curnow, Tu. Th. S.; H. T. Griffiths, M. W. F. Daily, between 9 and 3<br>*In- and Out-Surgeons*, Messrs. W. J. Smith, G. R. Turner. Daily, between 9 and 3 |
| **French Hospital,** 10, Leicester Place, Leicester Sq., W.C. Sec., Mr. E. Rimmel<br>Free to urgent cases and to all foreigners speaking French | *In-Physician*, Dr. Vintras, Tu. S. 10<br>*In-Surgeons*, Sir W. MacCormac, Tu. 9; Mr. J. Késer, W. F. 10<br>*Out-Physician*, Dr. Vintras, Tu. S. 10<br>*Out-Surgeons*, Messrs. H. de Méric, M. Th. 10; J. Késer, W. F. 10 |
| **German Hospital,** 113, Dalston Lane, E. Sec., Mr. C. Feldmann<br>Free to natives of Germany, and others speaking German, and to all accidents and emergencies. Others by Governors' letters | *In-Physicians*, Drs. Weber, M. T. 2; Port, Tu. F. 9 a.m.<br>*In-Surgeons*, Drs. Lichtenberg and Burger, W. S. 2<br>*Out-Physicians*, Drs. Tuchmann, Tu. W. 2; Ludwig, M. Th. 2; Keller, Tu. W. F. 2; Philippi, M. Th. F. 2 (*Men*, Tu. Th.; *Women*, M. W. F.) |
| ——— Eastern Branch, 49, Mansell Street, Aldgate, E. | *Out-Physician*, Dr. C. Harrer, Tu. F. 8 to 10 a.m. |
| ——— Western Branch, 259, Oxford Street, W. | *Out-Physician*, Dr. M. Castaneda, M. T. 8 to 10 a.m. |
| **Great Northern Central Hospital,** Caledonian Road, N. Sec., Mr. W. T. Grant<br>Free to all, without letters of recommendation | *In-Physicians*, Drs. Cholmeley, Cook, and Burnett, M. Tu. W. Th. F. 2<br>*In-Surgeons*, Messrs. W. Adams, W. Spencer Watson, and J. Macready, M. Tu. W. Th. F. 2<br>*Out-Physicians*, Drs. Beale, M. Th. 2; Beevor, Tu. 2, S. 10<br>*Out-Surgeons*, Messrs. Lockwood, M. Th. 2; Makins, Tu. F. 2 |
| **Guy's Hospital,** S. Thomas's Street, S.E. Supt., Dr. C. J. Steele<br>Patients are admitted, if beds are available, without letters of recommendation, on application at the Hospital. Out-patients are seen free without letters of recommendation | *In-Physicians*, Drs. Pavy, Tu. F.; Pye-Smith, M. Th. S.; Taylor, M. Tu. Th. F. Hour, 1.30<br>*In-Surgeons*, Messrs. Bryant, M. Th. Durham, M. Th. F.; Howse, W. S.; D. Colley, M. Th. Hour, 1.30<br>*Out-Physicians*, Drs. Carrington, W.; Hale White, M.; Goodhart, M. Tu. Th. F. Hour, 12.30<br>*Out-Surgeons*, Messrs. Lucas, Th.; Golding Bird, S.; Jacobson, W.; Symonds, M. Hour, 12.30 |
| **King's College Hospital,** Portugal Street, W.C. Sec., Mr. T. Mosse Macdonald<br>Accidents and urgent cases free at all times. Other cases by Governors' letters, but where not obtainable, application may be made to the Secretary | *In-Physicians*, Drs. Beale, Duffin, Yeo, Tu. W. F. 1.30; Tu. W. F. S. 2<br>*In-Surgeons*, Mr. Wood, Sir J. Lister, Messrs. H. Smith, W. Rose. Daily, 1.30<br>*Out-Physicians*, Drs. Ferrier, J. Curnow, N. I. C. Tirard. Daily, 1.<br>*Out-Surgeons*, Messrs. W. Rose, W. Cheyne, A. B. Barrow. Daily, 1. |
| **London Hospital,** Whitechapel Road, E. Sec., Mr. A. H. Haggard<br>Accidents and urgent cases free; out-patients for dental and maternity departments need no recommendation; other cases by subscriber's order. Out-patients are required to attend only once, unless otherwise directed by the Physician or Surgeon | *In-Physicians*, Drs. Mackenzie, Tu. F.; Down, Tu. F.; H. Jackson, M. Th.; Sutton, M. Th.; Fenwick, Tu. F. 1.30<br>*In-Surgeons*, Messrs. Rivington, Tu. F.; Couper, Waren Tay, M. Th.; McCarthy, W. S.; Treves, Tu. F. Hour, 1.30<br>*Out-Physicians*, Drs. Sansom, Th.; M. Ralfe, M. Th.; Turner, Tu. F.; Warner, Tu. F.; Gilbart Smith, W. S.; J. Anderson, W. S. Hour, 1.30<br>*Out-Surgeons*, Messrs. Mansell-Moullin, M. Th.; Eve, M. W.; Reeves, Tu. S.; H. Fenwick, Tu. F. Hour, 1.30 |
| **London Temperance Hospital,** Hampstead Rd.<br>For treatment without alcohol. By letter or small payment | *In- and Out-Physicians*, Drs. J. Edmunds, M.; J. J. Ridge, Tu. F. Hour, 1.30<br>*In- and Out-Surgeons*, Mr. A. P. Gould, Th. 2.30 |
| **Metropolitan Free Hospital,** Kingsland Road, N. Sec., Mr.<br>Admission free to the sick poor, without any letter of recommendation. Urgent cases at all times | *In- and Out-Physicians*, Drs. Drysdale, Tu. F. 12; J. G. Dudley, W. S. 12; Fotherby, M. Th. 12; H. H. Tooth, Tu. F. 9; A. Haig, W. S. 9; A. Davies, M. Th. 9<br>*In- and Out-Surgeons*, Messrs. E. J. Chance, W. S. 9; Goodsall, Tu. F. 9; Walsham, M. Th. 9; A. A. Bowlby, F. 9; S. Paget, Th. 9; C. J. Thompson, daily, 9 |

| Hospitals and Terms of Admission. | Physicians, Surgeons, and Medical Officers, with Days and Hours of Attendance. |
|---|---|
| **Middlesex Hospital,** Berners Street, W. Sec. and Supt., Mr. A. O. D. Bartholeyns<br>Urgent cases admitted at all times; other cases by ticket. All medical cases must, in the first instance, be seen by the Resident Medical Officer, who attends at the hospital daily, at 11 a.m. | *In-Physicians*, Drs. Cayley, M. Th.; Coupland, Tu. F.; D. Powell, M. Th.; Finlay, Tu. F. Hour, 1.30<br>*In-Surgeons*, Messrs. Hulke, M. Th.; Lawson, M. Th.; Morris, Tu. F. Hour, 1.30<br>*Out-Physicians*, Drs. Fowler, Tu. F. 3.30; Biss, M. Th. 1.30; Pringle, W. S. 1.30<br>*Out-Surgeons*, Messrs. Andrew Clark, M. 1.15 (*Men*); Th. 1.15 (*Women*); Pearce Gould, F. 1.30 (*Men*); Tu. 1.30 (*Women*); J. B. Sutton, W. S. 9 |
| **Miller Hospital and Kent Dispensary,** Greenwich, S.E. Hon. Sec., Mr. W. Bristow. | *Physicians*, Drs. T. Creed, C. H. Hartt, G. Willes<br>*Surgeons*, Messrs. T. Moore, J. Poland, E. Clarke |
| **North-West London Hospital,** 18, Kentish Town Road, N.W. Sec., Mr. A. Craske<br>By subscribers' tickets or payment, but accidents and urgent cases free at all times | *In- and Out-Physicians*, Drs. W. C. Hood J. Shaw, Edwarde H. Campbell. Daily 1.30<br>*In- and Out-Surgeons*, Messrs. F. Durham Collier, Jennings, J. Black. Daily, 1.30 |
| **Ospedale Italiano,** 41, Queen Square, Bloomsbury. Sec., Sig. L. Bonacina<br>Free to all, but Italians have preference | *In- and Out-Physicians*, Drs. M. Castaneda, Tu. F. 10 to 11; B. Sassella, M. Th. 10 to 11; Mr. J. W. B. Steggall, W. S 4 to 5 |
| **Poplar Hospital,** 303, East India Dock Road, E. Sec., Lt.-Col. Feneran<br>Free to accidents and emergencies | *In-Surgeons*, Messrs. F. M. Corner, M. Brownfield, and Resident Surgeons<br>*Out-Surgeons*, Dr. J. D. Grant, W. S; Mr. T. E. Bowkett, Tu. F. 12 to 2 |
| **Royal Free Hospital,** Gray's Inn Road, W.C. Sec., Mr. J. S. Blyth<br>Admission free to the sick poor, without any letter of recommendation | *In- and Out-Physicians*, Drs. Cockle, M. Th.; Sainsbury, Tu. F.; West, W. S. Hour, 1<br>*In- and Out-Surgeons*, Messrs. Rose, M. Th.; W. Gant, Tu. F.; Barrow, W. S. Hour, 1 |
| **St. Bartholomew's Hospital,** Smithfield. Clerk, Mr. W. Cross<br>Admission free, without letter or ticket. Accidents and other urgent cases admitted at any time; ordinary cases of disease daily, between 9 and 10. Patients are seen for Electrical treatment on M. Tu. Th. and F. 1.30 | *In-Physicians*, Drs. Andrew, Church, Gee, Sir D. Duckworth. Daily, 1.30<br>*In-Surgeons*, Messrs. Savory, T. Smith, Willett, Langton, M. Baker. Daily. 1.30<br>*Out-Physicians*, Drs. Hensley, M. Th. 11; Brunton, Tu. F. 11; Legg, W. S. 11; N. Moore, Tu. W. Th. S. 9<br>*Out-Surgeons*, Messrs. Marsh, Tu. F. 12; Butlin, M. Th. 12; Walsham, W. S. 12; Cripps and Bruce Clarke, daily, 9<br>*Casualty Physicians*, Drs. Davies, Tu. W. F. S.; O. Browne, M. Tu. Th. F.; Nias, M. W. Th. S. Hour, 9 |
| **St. George's Hospital,** Hyde Park Corner, S.W. Sec., Mr. C. L. Todd<br>Free to accidents and emergencies. Other in-cases by Governor's or subscriber's letter, but, in necessitous cases, this recommendation is not strictly insisted on. Letters are not required for out-patients. Vaccination on Th. at 11 | *In-Physicians*, Drs. Wadham M. F.; Dickinson, M. Tu. Th. F.; Whipham, Tu. W. S.; Cavafy, Tu. S. Hour, 1<br>*In-Surgeons*, Messrs. Holmes, M. Th. F. 1, W. 1.30; Rouse, M. Th. F. 1, W. 1.30; Haward, Tu. Th. S. 1, W. 1.30<br>*Out-Physicians*, Drs. Ewart, M. F.; Owen, Tu. S. Hours, 10.30 to 12<br>*Out-Surgeons*, Messrs. Bennett, M. F.; Dent, Tu. S. Hours, 10.30 to 12<br>*Men*, F. S.; *Women*, M. Tu. |
| **St. Mary's Hospital,** Cambridge Place, Paddington, W. Sec., Mr. Thomas Ryan<br>In-patients are admitted by letter of recommendation. Urgent cases and accidents free at all times. Out-patients require a letter. Electrical treatment Tu. and F. 2 | *In-Physicians*, Sir Edward Sieveking, Tu. F. 1.45; Drs. Broadbent, M. Th. 1.45; Cheadle, W. 1.45, S. 9.30<br>*In-Surgeons*, Messrs. Norton, M. Th. 1.45; Owen, Tu. F. 1.45; H. Page, W. S. 1.45<br>*Out-Physicians*, Drs. D. B. Lees, W. S.; S. Phillips, M. Th; R. Macguire, Tu. F. Hour, 1.30<br>*Out-Surgeons*, Messrs. W. Pye, M. Th.; A. J. Pepper, Tu. F.; A. Q. Silcock, W. S. Hour, 1.30 |
| **St. Thomas's Hospital,** Westminster Bridge Road, S.E. Steward, Mr. F. Walker<br>Accidents and emergencies free at all times. Other cases by Governor's letter, but, where unobtainable, application should be made to the Steward by letter, or personally between 10 and 4. Vaccination on W. at 11.30 | *In-Physicians*, Drs. Bristowe, Tu. F.; Stone, M. Th.; Ord, M. Th.; J. Harley, Tu. F.; Gervis, Tu. F. Hour, 2<br>*In-Surgeons*, Messrs. Sydney Jones, Tu. F.; Croft, M. Th.; Sir W. MacCormac, M. Th. Hour, 2<br>*Out-Physicians*, Drs. Payne, Tu. F.; Sharkey, M. Th.; Gulliver, W. S. Hour, 1.30<br>*Out-Surgeons*, Messrs. Clutton, Tu. F.; Anderson, W. S.; Pitts, M. Tu. Hour, 1.30 |
| **Tottenham Training Hospital,** Tottenham, N. Director, Dr. Laseron<br>Free. Accidents and emergencies admitted at all times | *In-Physician*, Dr. Rasch, Tu. F. 3 to 5<br>*In-Surgeons*, Drs. Lichtenberg, M. Th. 3 to 5; E. H. May, M. Th. 3 to 5<br>*Out-Surgeon*, Dr. Lloyd Smith, M. W. 11 to 2; *Men* only, W. 7 to 8 p.m. |

### AMUSEMENTS WITH PRIZES, FOR ALL AGES.

### SPECIAL NOTICE.

DURING the past year we have offered upwards of 30 guineas in prizes, of which upwards of twelve have been for children, who have come forward in considerable numbers. For some reason the competitors for the remaining twenty pounds have been comparatively so few as to make it doubtful whether to continue these prizes beyond the 15th inst., when a new series is due to commence. We are disposed to continue the experiment for another year, if there is any general wish amongst the readers of THE HOSPITAL that this shall be done. Few at present appear to realise that it is possible to earn three guineas a quarter and five guineas once a year under the present scheme. We shall therefore give an extra prize of one guinea to the correspondent who sends the names and addresses of the greatest number of subscribers, old or new, who will undertake to enter for the prizes offered to men and women during the ensuing year, and a further prize of one guinea for the best suggestion for increasing the interest in these competitions. These prizes are subject to the condition that at least twenty competitors enter for each of them, and that the lists and suggestions reach us not later than Monday, the 17th inst. All answers should be addressed to the Editor, THE HOSPITAL, Norfolk House, Norfolk Street, London, W.C., and have distinctly written in the left-hand corner of the envelope the words "New Prize Competition." The *nom de plume* of all competitors for these special prizes will be published on 22nd October with the results, and our decision for the coming year.

### FOR MEN AND WOMEN.

### Quarterly Prize Competition.

Began 23rd July; ends 15th October, 1887.

**A Prize of Three Guineas**

will be awarded to the Competitor who shall have sent in the largest number of correct or best answers.

**A Prize of Five Guineas**

to the competitor who shall have sent in the largest number of correct or best answers during the twelve months.

### No. 23. Double Acrostic.

Dullest and driest labour of man's brain.
Yet blended with a name few can attain:
And, added to the ranks my last commands,
A useful drudge, in every house it stands.

1. A word most difficult for some to speak,
   And yet, without such power all men are weak.
2. Barbarous, yet valiant, well they held their land,
   When Roman conquerors stepped on British strand.
3. An ancient race, whose valour minstrels sound.
   Once spread through Europe, and maintained their ground.
4. Past, present, future—all my power confess.
   The schoolboy's watchword, pianist's distress.
5. The marked condition which some spirits prove,
   Foul foe to peace, to comfort, and to love.
6. A Russian port much guarded by the state,
   In corn and wool its exports great.
7. Rough and unpolished, of uncertain weight,
   But still of value, be it small or great.
8. A benediction of such common use,
   We're apt its sacred meaning to abuse.
9. A happy boundary placed by Nature's hand,
   Checking dissensions between land and land.
10. The time of old, tradition of past years,
    That lore of history welcome to all ears.

### No. 24. Anagram.

I'm a widely-famed country—the home of the rose,
Where bliss is the voice of all nature;—transpose,
And you'll find that of which very few are afraid,
But too often the spring of our actions is made.
Transpose me again, till you bring to your view
What all, when they strive to be mightier, do.

### Answers.

No. 19. DOUBLE ACROSTIC.

| | | |
|---|---|---|
| S | po | T |
| U | s | U |
| L | ea | R |
| T | uc | K |
| A | ccolad | E |
| N | anc | Y |

Correct answers have been received from Boss, Mater, Condorcet, Gretta, Brownie.

No. 20. AMUSEMENTS FOR CONVALESCENTS.

The papers received on the above subject contain some thoughtful and practical suggestions for alleviating the tedium inseparable from the period of convalescence. On a future occasion, we hope to reproduce some of the ideas furnished by our correspondents.

Answers have been received from Ellice, Brownie, Aralc, Mater Gretta, Perseverance, Condorcet.

### THE CHILDREN'S CORNER.

PRIZES.
FOR MOST CORRECT ANSWERS TO PUZZLES.
One Pound per Month.
FOR BEST LITTLE LETTERS.
Ten Shillings per Quarter.
A PRIZE OF THREE GUINEAS
to the Competitor who shall have sent in the largest number of correct or best answers during the twelve months.

### Puzzles—Second Week.

No. 5. BURIED RIVERS. 1. Do you see the rainbow, Edgar? 2. Lawn tennis is a very good game. 3. Did you go to Derbyshire this summer?

No. 6. PYRAMID PUZZLE. With eight pieces of paper of the size of fig. *a*, four of fig. *b*, and four of fig. *c*, and of proportionate sizes, form a perfect square.

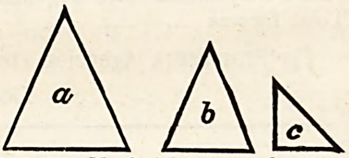

No. 7. BIOGRAPHICAL CHARADE. My first is composed of two consonants and a vowel; my second is made with plaster-of-paris; my third is the beginning of error; and my whole a royal earl who lost his life in a rebellion against Edward II.

No. 8. CHARADE.

My first in revolutions makes it way,
My next's the tomb of Saxon chivalry,
My whole with two legs manages so ill,
It only uses them when sitting still.

### Letters—Second Week.

Next week "Your Favourite Poets, and their Poems," will be the letter-subject.

### Results of Thirteenth Week—Puzzles.

No. 50. ARITHMETICAL PUZZLE. The man had, of course, seven sheep, because

$$7+7+3\tfrac{1}{2}+2\tfrac{1}{2}=20=\text{one score.}$$

No. 51. Captain Forbes (four B's) sent his forces (four C's) to the East Indies (east in D's).

No. 52. GEOGRAPHICAL ACROSTIC.

| | | |
|---|---|---|
| W | ell | S |
| A | pri | L |
| L | ouisian | A |
| E | f | T |
| S | ein | E |

The country is Wales, famous for slate.

No. 53 TRANSPOSITION. The *R. Wear* (in Durham), transposed becomes the *Town Ware* (in Hertfordshire). This question puzzled many of the competitors. Several cleverly guessed the *R. Tone*, which, transposed, gives *Eton*. As this is only a small river, I hope all who gave it to me know where it is to be found.

*All right*—Mount Edgcumbe, Toby, A. G. S. McCulloch, Scrooge, Umslopogaas; *Three right*—Poodle, Agatha, A. C. K.; *Two right*—The Eda, Skye, Ossian, Fern; *One right*—Princess Ida.

### Letters.

Three marks each:—The Eda, Toby, Socrates, Princess Ida. Two marks each—Skye, Mount Edgcumbe, Ossian.

### Notices to Correspondents.

POODLE.—Thank you very much for the riddle

21, CHAMPION PARK, LOWER SYDENHAM.—Please send your full name, and choose a shorter *nom de plume.*

### Rules.

All answers must be addressed to THE PRIZE EDITOR, THE HOSPITAL, Norfolk House, Norfolk Street, Strand, W.C., not later than the *first* post on Friday next. The full name and address must in all cases be given, but a *nom de plume* may be added for publication. Only one side of the paper should be written on.

No one will be permitted to win the Monthly or Quarterly Prizes twice in any one year, the annual prizes being offered especially to prevent this.

